# Characteristics and optical properties of atmospheric aerosols based on long-term AERONET investigations in an urban environment of Pakistan

**DOI:** 10.1038/s41598-024-58981-0

**Published:** 2024-04-12

**Authors:** Bahadar Zeb, Khan Alam, Rehana Khan, Allah Ditta, Rashid Iqbal, Mohamed Farouk Elsadek, Ahsan Raza, Mohamed Soliman Elshikh

**Affiliations:** 1 Department of Mathematics, Sheringal Dir (Upper), Shaheed Benazir Bhutto University, Khyber Pakhtunkhwa, Pakistan; 2 Department of Physics, University of Peshawar, Khyber Pakhtunkhwa, Pakistan; 3 Department of Physics, Higher Education Colleges, Govt. of Khyber Pakhtunkhwa, Peshawar, Pakistan; 4 Department of Environmental Sciences, Shaheed Benazir Bhutto University Sheringal, Dir (U), Khyber Pakhtunkhwa, 18000 Pakistan; 5 School of Biological Sciences, The University of Western Australia, 35 Stirling Highway, Perth, WA 6009 Australia; 6 Department of Agronomy, The Islamia University of Bahawalpur, Bahawalpur, 63100 Pakistan; 7 Department of Biochemistry, College of Science, King Saud University, P.O. 2455, 11451 Riyadh, Saudi Arabia; 8 Institute of Crop Science and Resource Conservation (INRES), University of Bonn, Bonn, Germany; 9 Department of Botany and Microbiology, College of Science, King Saud University, 11451 Riyadh, Saudi Arabia; 10 Leibniz Centre for Agricultural Landscape Research (ZALF), Eberswalder Straße 84, 15374, Müncheberg, Germany

**Keywords:** AERONET, Aerosols, Metropolitan, Clean continental, Aerosol optical depth, Angstrom exponent, Climate sciences, Atmospheric science

## Abstract

Radiative balance, local climate, and human health are all significantly influenced by aerosol. Recent severe air pollution over Lahore, a city in Pakistan calls for more thorough research to determine the negative impacts brought on by too many aerosols. To study regional aerosol characteristics and their differences from various aspects, in-depth and long-term (2007–2020) investigations of the columnar aerosol properties over the urban environment of Lahore were carried out by using AERONET data. The Aerosol Optical Depth (AOD_400_) and Angstrom Exponent (AE_400–870_) vary from low values of 0.10 to a maximum value of 4.51 and from 0.03 to 1.81, respectively. The huge differences in the amount of AOD_440_ as well as AE_440–870_ show the large fluctuation of aerosol classes because of various sources of their emission. During the autumn and winter seasons, the decreasing trend of the optical parameters of aerosols like Single Scattering Albedo (SSA) and Asymmetry Parameter (ASY) with increasing wavelength from 675 to 1020 nm indicates the dominance of light-absorbing aerosols (biomass burning (BB) and industrial/urban (UI). Due to the long-distance dust movement during spring, summer, and autumn, coarse mode particles predominated in Lahore during the study period. Dust type (DD) aerosols are found to be the dominant one during spring (46.92%), summer (54.31%), and autumn (57.46%) while urban industry (BB/UI) was dominant during the winter season (53.21%). During each season, the clean continental (CC) aerosols are found to be in negligible amounts, indicating terrible air quality in Lahore City. The present research work fills up the study gap in the optical properties of aerosols in Lahore and will help us understand more fully how local aerosol fluctuation affects regional climate change over the urban environment of Lahore.

## Introduction

Aerosols encompass solid or liquid particles suspended in the atmosphere, comprising industrial waste, vehicle exhaust, and globally dispersed dust, with a recognized significant impact on climate change^1^. The Earth's energy equilibrium may be affected by the absorption or scattering of these aerosols^2,3^. The various aerosols in our atmosphere play a crucial role in processes such as precipitation, the land-atmospheric water cycle, and visibility constraints4. Globally, the substantial effects of aerosols cannot be ignored^4^. The temporal and spatial fluctuations of aerosols have noteworthy consequences on specific climatic conditions, and consequently, on human health^2^. Further research is necessary to comprehensively understand the diverse optical, physical, and chemical characteristics of aerosols and their various climatic implications^1,5^. Given the pivotal role of aerosols in climate change, it is essential to explore their various crucial optical parameters, including Aerosol Optical Depth (AOD), Phase Function (PF), Single Scattering Albedo (SSA), Asymmetry Parameter (Asy), as well as scattering and extinction parameters^6,7^.

Generally, AOD exhibits larger values in the hot season and decreases during the dry and cold seasons across diverse locations of Asia, as indicated by geographical and temporal variations in aerosol content^8–10^. Various aerosol properties were utilized to differentiate between distinct types of aerosols^7,11^. Both AOD and Angstrom Exponent (AE) are crucial parameters for comprehending diverse aerosol types, their origins, and their distribution in the atmosphere^12^. AE serves as a valuable metric for assessing aerosol properties such as formation, growth, size, and effective radius^4,13^.

The size of the aerosol particle, its effective radius, and its production and growth mechanisms can all be learned via AE^4,13^. By combining AERONET-derived AAE, EAE, and SSA, cluster analysis is used to produce several aerosol classifications^13–17^. Pakistan is experiencing significant environmental issues because of rapid industrial growth and urbanization^18–23^ with big cities like Karachi, Lahore, and Peshawar, being particularly hard hit^24–27^. These significant environmental problems have detrimental effects on both transportation^28,29^ and human health^3,30^. Various aerosol loadings that cause long-lasting heavy haze, deteriorated visibility, and other pollution events have been identified in many studies. A recent study^31–34^ attributed around 80% (by mass) of the total transport emissions (PM_10_/PM_2.5_) in Lahore to motorcycles and scooters. Numerous studies have revealed a variety of aerosol loadings that result in persistently high haze, declining visibility, and other pollution occurrences. The varying climatic circumstances may also be to blame for all these issues, in addition to the rising anthropogenic activities^35,36^.

Based on these results, the main objective of this research is to investigate the long-term (2007–2020) climatology of aerosol properties and affiliated effects of different aerosol types over the megacity of Pakistan. To distinguish and categorize different aerosols, their numerous sources, and transformation mechanisms, long-term analysis of aerosol parameters is required^10,37^. Previously, numerous methods were used to distinguish between various forms of aerosols and their effects on regional climate^17,38^. Atmospheric forcing obtained from ground-based sensors and their absorption capability are useful techniques to characterize the heterogeneity of atmospheric aerosols^30,39–41^^.^ The seasonal classification of aerosols is also carried out to monitor the dominating kind and its effects on the climate and monsoon circulation. AOD and AE clustering have been the subject of numerous studies to classify and measure various aerosols^39–42^, while others have involved EAE and AAE clustering^15,17,43–45^. Relatively few studies have been recorded to characterize the numerous aerosols in Pakistan. This indicates that more research and analysis are still needed. This paper employs a clustering technique to examine in-depth details of distinct aerosols over Lahore by using long-term AERONET data from 2007 to 2020. The dominating aerosols and their seasonal changes over the site are investigated using a variety of aerosol characteristics. Conducting this type of analysis is essential to distinguish between different aerosol types, their distribution patterns, and their primary sources by considering a range of features. The present study seeks to offer an updated account of the optical characteristics of aerosols, their daily and seasonal variations, and their clustering (aerosol types) in the urban atmosphere of the mega-city, Lahore, Pakistan. Further, the findings aim to shed light on the mechanisms contributing to aerosol pollution in Lahore. To evaluate the quality of the retrieved data, numerous studies^31,32,46,47^ have been conducted in the recent past for comparison of AERONET with the ground base (CARSNET, CARE-China, CSHNET), Model (MERRA-2) and satellite data (MODIS, MISR, CALIPSO) for short to long term study period and found it compatible in all the cases, so it (Data Comparison/validation) has been omitted from the present work to prevent redundancy.

## Results and discussion

### ***Monthly variation in AOD***_***440***_*** and AE***_***440–870***_

**Figure 1:**
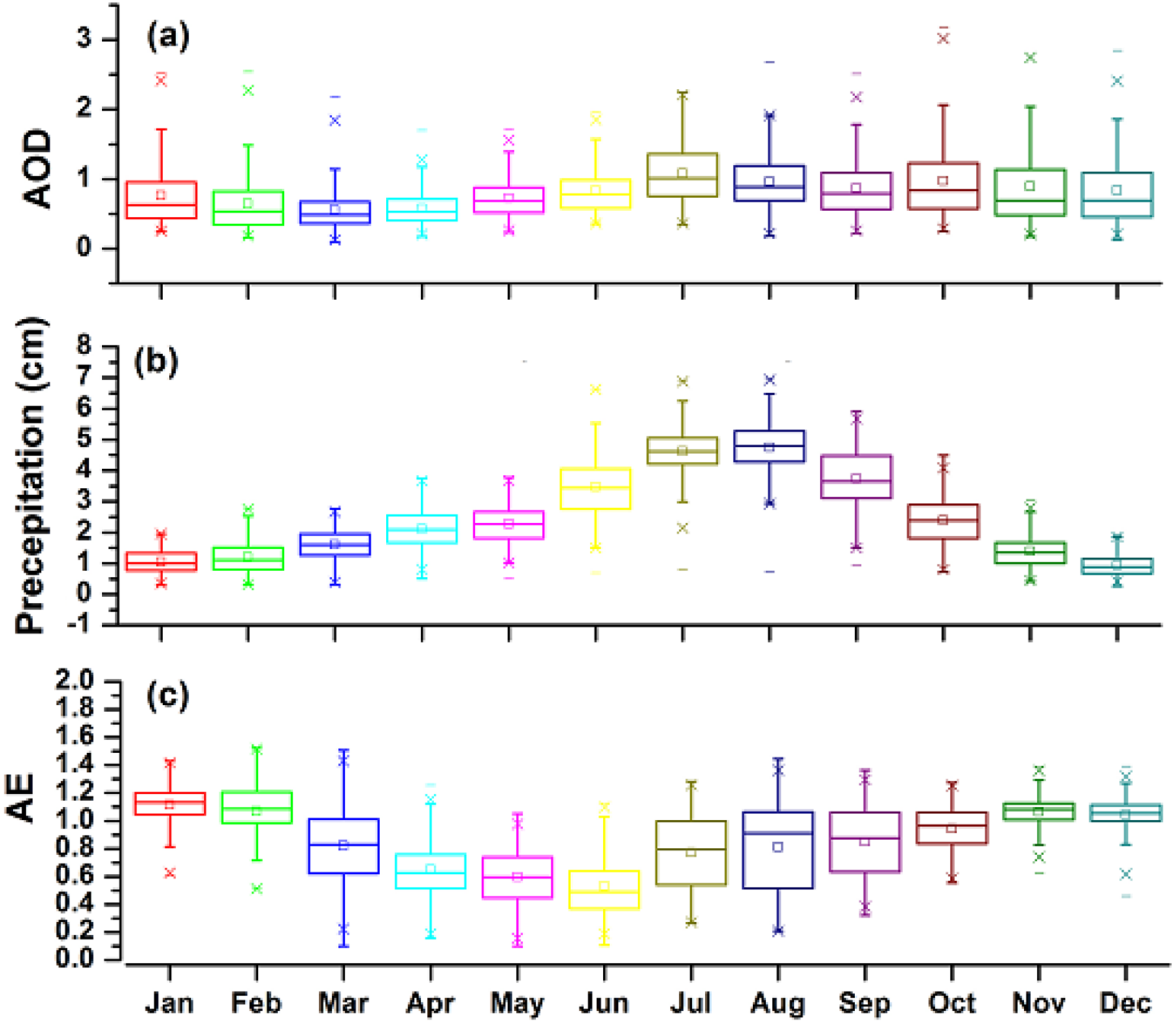
Monthly fluctuations in (**a**) AOD at 440 nm (**b**) Precipitation (cm) (**c**) AE at 440–870 nm. The first and third quartiles, respectively, represent the bottom and upper bounds of each box, while the median is represented by the central line. The vertical lines that stretch out from the box represent the distribution's spread, and their length is equal to 1.5 times the difference between the first and third quartiles. The geometric means are denoted by asterisk symbols.

AOD and AE provide information about atmospheric columnar aerosol load and aerosol size, respectively. The monthly variation of AOD_440_, AE _440–870_ is shown in Fig. 1. During the whole study period, AOD_440_ varied from a minimum value of 0.10 to a maximum value of 4.51. Low values of AOD_440_ i.e. < 0.01 indicate a clean air background, while its high values i.e. > 0.5 or even > 0.1 show harsh atmosphere aerosols load because of the effects of anthropogenic pollutants or transported dust^15^. The strong fluctuation in the AOD_440_, and AE _440–870_ values during the study period represents regular changes in the aerosol types in Lahore brought on by its diverse emission sources.

The seasonal average AOD and AE are over Lahore as indicated in Fig. 1. The maximum AOD is found during summer (0.95 ± 0.39) with corresponding moderate AE (0.79 ± 0.37). This suggests that coarse mode particles associated with the mixing of anthropogenic contaminants and transportable dust characterize aerosols throughout this season^48,49^. On the other hand, the lowest AOD (0.61 ± 0.28) with the corresponding lowest AE (0.77 ± 0.30) was investigated during spring seasons. During the spring season, the nature of the aerosol particles seems to be similar to in the summer season but with the lowest aerosol burden. The moderate value of AOD _400_ (0.75 ± 0.47) with the highest value of AE (1.25 ± 0.21) during the winter season implies that the aerosol particles are in the fine mode, which is created by transported aerosols, vehicle exhaust, and industrial emissions^50^. Similarly, conditions like a high value of AOD (0.91 ± 0.54) and high AE (1.10 ± 0.25) during autumn are favorable for fine-mode aerosol particles.

Winter season faces severe pollution and is found in more stagnant weather conditions as compared to other seasons^51^. The AE values are found to be consistent at around 1.2 throughout the winter, indicating the dominance of fine-mode particles across the research area^52^. The winter season in Lahore is very cold and dry and does not provide favorable conditions for the hygroscopic growth of atmospheric aerosols. Likewise, summertime has the lowest AE values, which can be attributed to strong winds (Fig. 1) that mix anthropogenic emissions from the local area with dust that has been carried^53^.

### Seasonal variation in single scattering albedo

**Figure 2:**
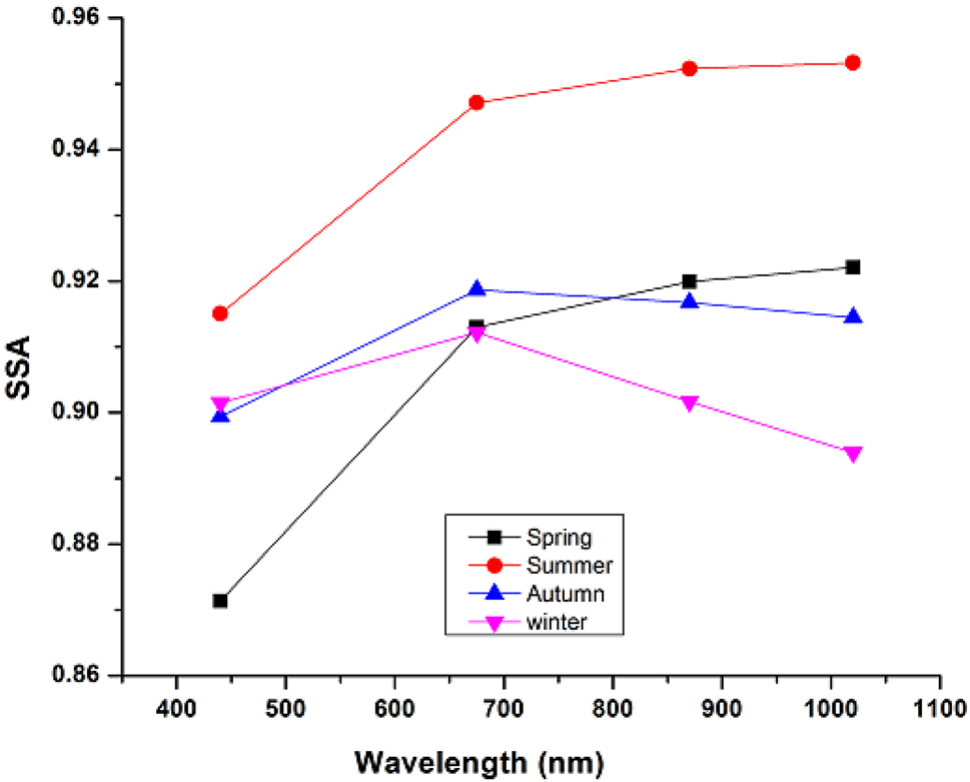
Single scattering albedo seasonal spectral fluctuation over Lahore, Pakistan during the study period (2007–2020).

Single Scattering Albedo (SSA) is the ratio of scattering to total solar radiation extinction and is the most important optical parameter for determining the scattering and absorption characteristics of aerosol particles^54^. The greater value of SSA_440_ (> 0.95) corresponds to weakly absorbing particles for example sulfate. On the other hand lower values of SSA_440_ (< 0.85) correspond to strongly absorbing particles i.e. black carbon/soot. The SSA value in the range from 0.85 to 0.90 and 0.90–0.95 corresponds to moderately absorbing and slightly absorbing aerosol particles, respectively^53^. The variations of SSA with a wavelength of solar radiation can be used for the understanding of prominent aerosol types i.e. dust, sulfate, and black carbon, and can further be used in combination with AE for the separation of atmospheric aerosol classes^54^. The SSA is obtained over Lahore at four wavelengths, 440, 675, 870, and 1020 nm, to examine aerosol fluctuations. Figure 2 displays the seasonal mean variation of the SSA. The overall mean values of SSA at 400 nm are found to be 0.90 ± 0.02 indicating moderately absorbing aerosols and is comparable with the results of the urban environment i.e. Xianghe (0.90) and less than Shanghai (0.91), Tongyu (0.91) and Harbin (0.93)^53,55–57^. The SSA_440_ during spring, summer, autumn, and winter are found to be 0.86 ± 0.03, 0.91 ± 0.03, 0.89 ± 0.02 and 0.89 ± 0.03, respectively. The variation in SSA values indicates various classes of aerosol particles during the study period over Lahore.

There are absorbing and scattering components in aerosol particles, as seen by the SSA_440_'s seasonal variations with wavelength changes ^52^. The increasing trend of SSA_440_ with wavelength during spring and summer indicates the presence of coarse particles. Conversely, the near-infrared region's declining SSA_440_ trend with increasing wavelength indicates the presence of absorbing aerosol particles. Other researchers in Pakistan, India, and China also reported spectral variation of SSA during various seasons^16,52,54,58^.

### Seasonal variation in asymmetry parameter

The Asymmetry Parameter (ASY) is an extremely important optical parameter that represents the angular distribution of solar light scattered by aerosol particles and is crucial in regulating aerosol radiative forcing, which in turn modifies regional climate^59^. ASY_440_, which is defined as the weighted average of the scattered phase function, is dependent upon the shape, size, and chemical composition of the aerosol particles. The values of ASY_440_ vary from − 1 (completely backscattering) to + 1 (completely forward scattering), with 0 representing symmetric scattering^54^.

**Figure 3:**
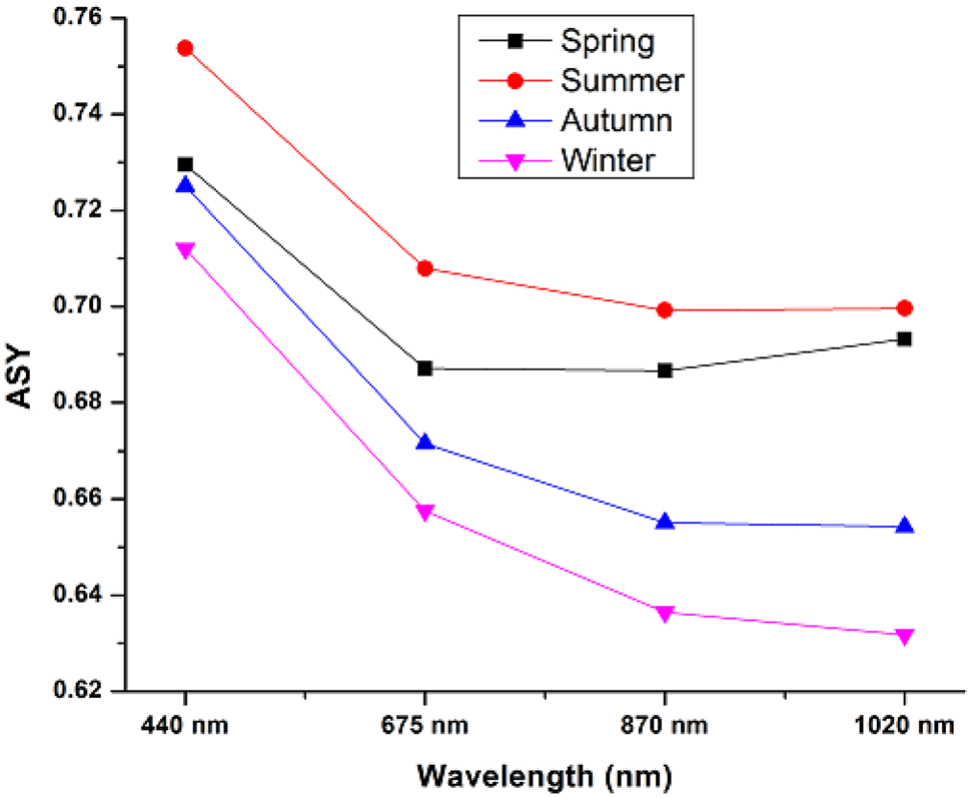
Asymmetry factor seasonal spectral fluctuation over Lahore during the study period.

The seasonal average variation of ASY_440_ with the wavelength of solar radiation during the study period over Lahore is shown in Fig. 3. The ASY440 during spring, summer, autumn, and winter are found to be 0.73 ± 0.02, 0.75 ± 0.01, 0.72 ± 0.02 and 0.71 ± 0.02, respectively. During winter and autumn seasons, the ASY_440_ decreases with wavelength and indicates strong wavelength dependency, which shows that the aerosol particles are absorbing. However, the ASY440 exhibits minimal wavelength fluctuation in the near-infrared region during the spring and summer, indicating the presence of transported coarse dust aerosol. Lahore experiences cool autumn and winter seasons, with AE 400–870 values greater than 1.0 suggesting the presence of fine aerosol particles. The values of ASY440 during autumn (0.72 ± 0.02) and winter (0.71 ± 0.02) are well in line with the values reported by D´Almeida et al.^60^, according to which the ASY440 values nearly equal to 0.72 indicates fine mode dry aerosol particles. The obtained results are similar to the results reported during the dusty days over different locations like Shirahama, SACOL, Noto, and Beijing^55,61^.

### Seasonal variation in volume size distribution

**Figure 4:**
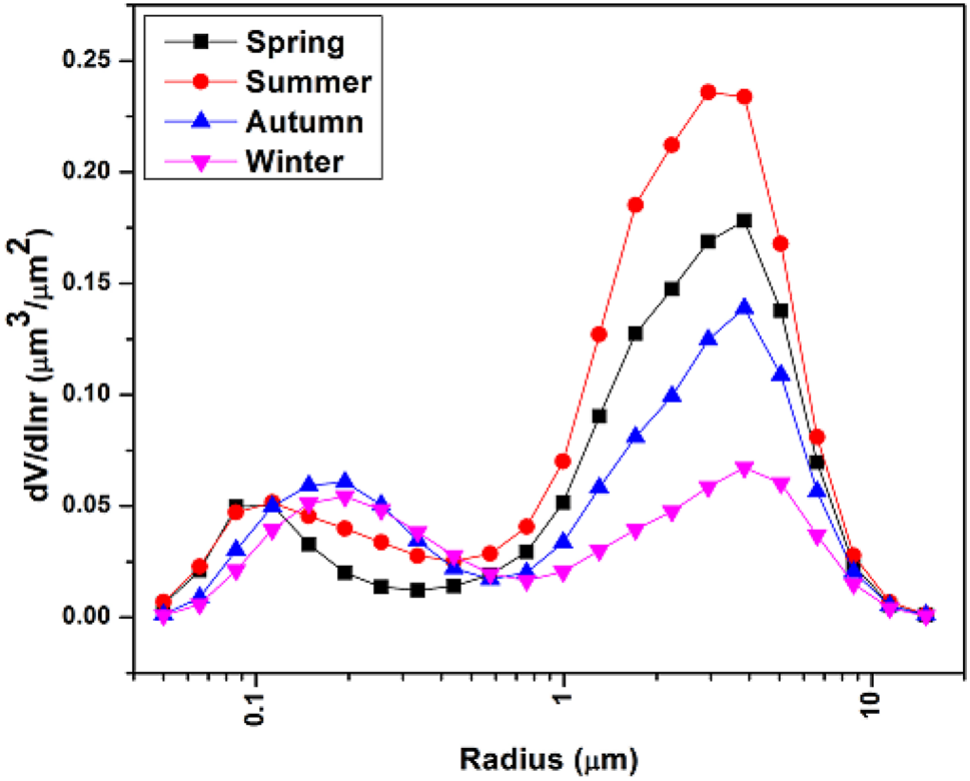
Seasonal variations in the volume charge distribution in Lahore, Pakistan during the research period (2007–2020).

The columnar aerosol size distribution, which is strongly related to AE and its changes, is a crucial factor in determining the aerosol optical characteristics^61^. Compared to the fine mode particles, which are mainly associated with anthropogenic activities like fuel combustion and vehicle exhaust, the coarse mode particles are more likely to consist of locally suspended dust that is carried over great distances by strong winds or released during building construction^56^. Figure 4 shows the seasonal mean of the aerosol volume size distribution over urban Lahore. The fine mode radius is found to be < 0.6 μm and the coarse mode radius to be > 0.6 μm, indicating that the aerosol volume size distributions have a bimodal logarithm normal structure. During spring and summer, the volume concentration of fine mode particles peaks at a radius of 0.11 μm, while in the autumn and winter, it peaks at a radius of 0.19 μm. Similarly, during spring, autumn, and winter the coarse mode, the peak occurs at radii of 3.9 μm, and during summer it occurs at 2.9 μm. During spring, the coarse mode particles dominate over urban Lahore mostly because of the presence of transport dust particles, which have comparable seasonal mean volume size distribution at the SACOL and Beijing sites^55,62,63^. Because of its geographic location, which is marked by flatter rain, a predominate wind that is acquainted with the west side of SACOL^55,62,63^, Harbin has frequently been influenced by the carried dust during the study period.

The presence of suspended snow crystals and long-range dust aerosols is suggested by the coarse mode particles, which are still predominant in the spring, autumn, and winter and have a larger coarse mode radius^55,62^. Winter has a slightly higher volume concentration of fine modes than other seasons. This suggests that heating causes anthropogenic emissions such as soot particles and that frequently occurring weather conditions that are detrimental to the diffusion of pollutants also cause aerosols to coagulate^54^.

### Determination of aerosol classes

Aerosol type can be determined using information on the optical and physical features of the aerosols' spectrum fluctuation. The most often used technique for differentiating between different types of aerosols is to combine AOD and AE, which stand for aerosol loading and aerosol size, respectively^54,64^.

**Figure 5:**
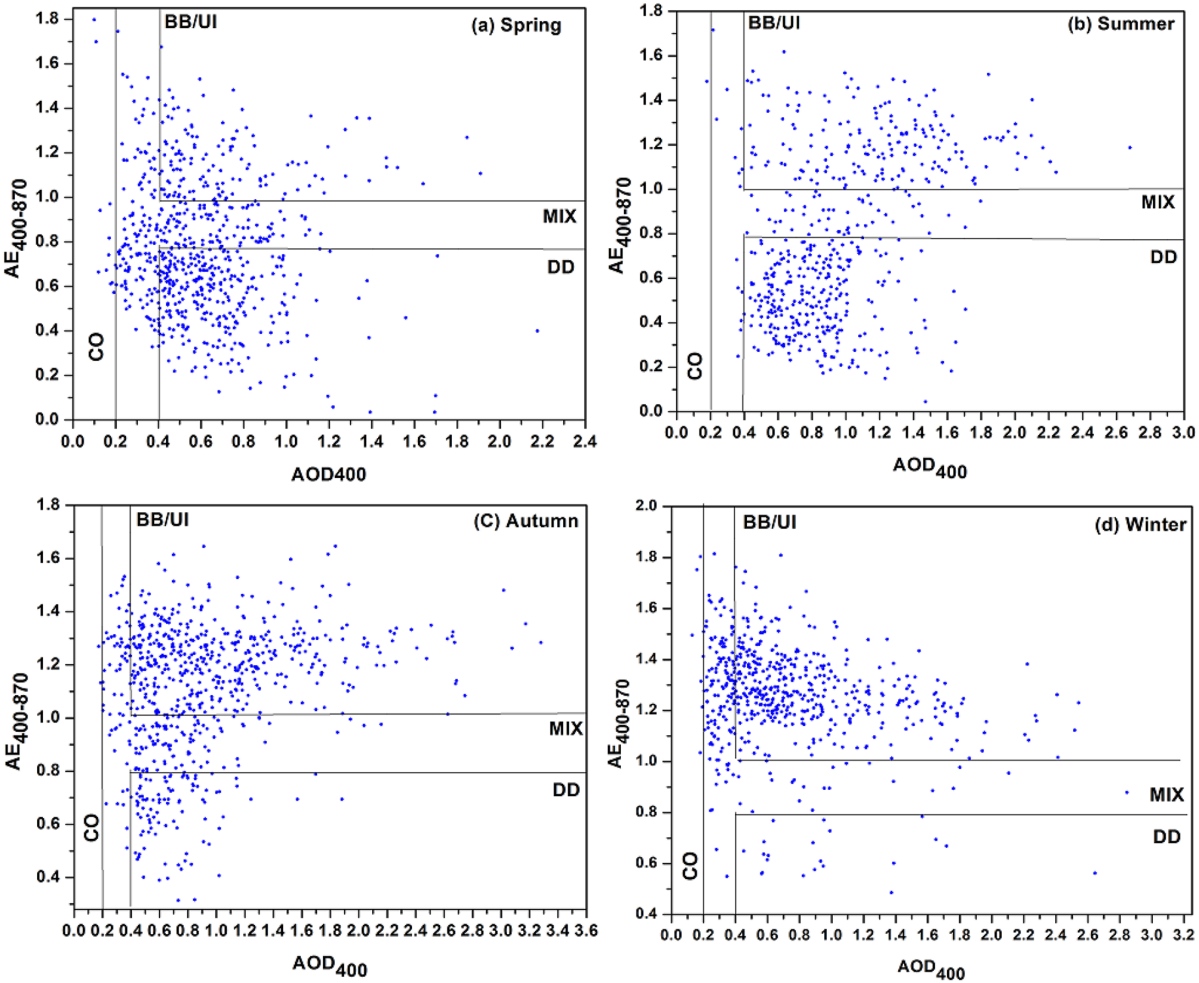
A scatter chart of the daily average values of AOD_440_ and AE_400-870_ shows the predominant aerosol classes over Lahore, Pakistan, in the (**a**) spring, (**b**) summer, (**c**) autumn, and (**d**) winter.

Figure 5 shows the scatter plot of the daily mean value of AOD _440_ and AE_440-870._ A broad range of AE _440–870_ values for low to high AOD _440_ demonstrates the great diversity in aerosol properties and also indicates that different forms of aerosol are mixing in the atmosphere above Lahore's metropolitan environment. To quantify the contribution of the main aerosol kinds, some threshold values should be considered before determining the aerosol types. Clean continental aerosols (CC) are defined as AOD _440_ < 0.2, representing the average conditions over Lahore, and long-range transported desert dust aerosols (DD) as AOD _440_ > 0.4 and less than 0.5 and AE _440–870_ < 0.8. Cases involving transportable biomass-burning aerosols or thick urban/industrial plumes (BB/UI) are taken into consideration for AOD_440_ > 0.4 and AE_440-870_ > 1.0, whereas cases that do not fit into any of the aforementioned categories are called mixed-type aerosols (MIX)^65,66^.

**Figure 6:**
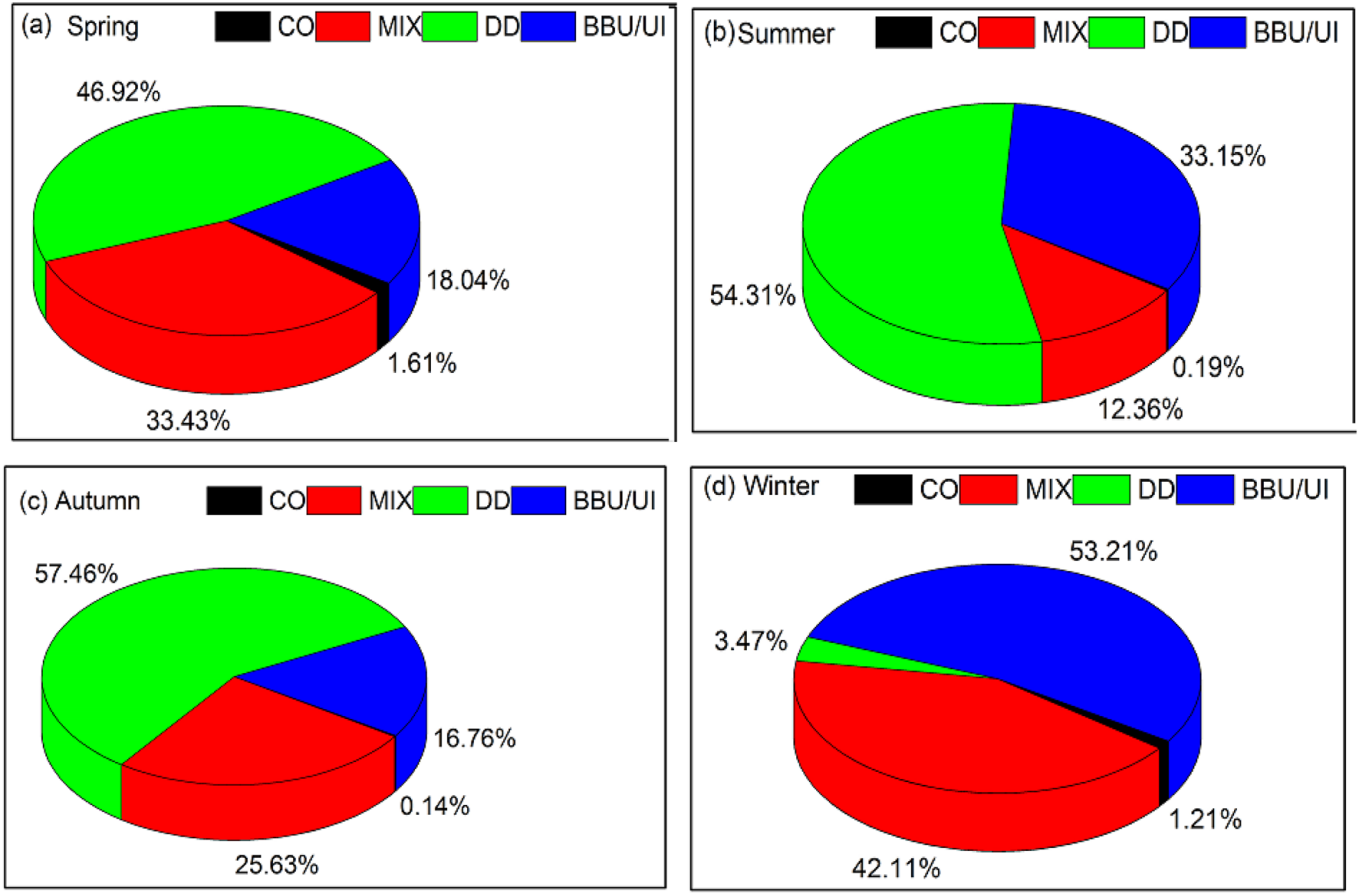
Percentage contribution of different types of aerosols during (**a**) spring (**b**) summer (**c**) autumn (**d**) winter.

The seasonal percentage contributions of four aerosol types over Lahore during the long-term study period are shown in Fig. 6. During the winter season, the BB/UI has the topmost contribution (53%) followed by MIX (42.1%) aerosols, it is likely that local and transported pollutants, such as the burning of agricultural crop residues, vehicle exhaust, and soot particles from heating, have a significant impact on the amount of anthropogenic aerosols that are present in Lahore. In addition, DD aerosols make up a sizeable component, accounting for a relatively high percentage i.e. 57%, 50%, and 39%, during autumn, summer, and spring, causing Lahore to frequently experience dust aerosols.

The fact that CC aerosols only make up very little i.e. 1.60% in the spring, 0.19% in the summer, 0.14% in the autumn, and 1.21% in the winter, indicates that Lahore's air quality is far worse than that of the majority of south Chinese cities (low aerosol load with AOD _440_ < 0.2)^52^. The results presented above mostly depend on the threshold used, and modifications to the threshold value may have a significant impact on the contribution^54^.

## Conclusions

Thirteen years of ground-based observations from AERONET over the urban environment of Lahore, Pakistan, from 2007 to 2020 were used to analyze regional aerosol optical features (AOD, AE, SSA, ASY, and VSD) and classify aerosol species. The values of AOD_440_ in four seasons i.e. spring, summer, autumn, and winter are found to be 0.61, 0.95, 0.91, and 0.74 with corresponding AE_440–870_ values of 0.77, 0.78, 1.10, and 1.25, respectively. The significant variation in both AE_440–870_ and AOD_440_ shows how frequently the types of aerosols over Lahore change. The SSA _400 nm_ in spring, summer, autumn, and winter are found to be 0.87, 0.91, 0.89, and 0.90, respectively with an overall average of 0.89 ± 0.03, indicating that the aerosols over Lahore are absorbing. Similarly, the mean ASY during spring, summer, autumn, and winter are found to be 0.72, 0.75, 0.72, and 0.71, respectively. The results show that the values of ASY decrease with increasing wavelength during the winter season showing that the particles are of absorbing nature. The summer and springtime near-infrared values of ASY exhibit a little reduction, indicating the impact of transported coarse-mode dust particles. Bimodal logarithmic behavior characterizes the aerosol volume size distribution, with a fine mode radius of 0.11 μm in the spring and summer and 0.19 μm in the autumn and winter. Likewise, the coarse mode radius of 3.9 μm was investigated during spring, autumn, and winter and 2.9 during summer. This implies that during the study period, fine and coarse-mode aerosols over Lahore were equally significant. Aerosol type discrimination results showed that during spring, summer, and autumn, DD aerosol predominated, as did BB/UI aerosol during winter, whereas CC aerosols made up a very small part, indicating poor air quality in Lahore city. Our knowledge of the local aerosol characteristics, regional movement, and their climatic consequences over Lahore would be enhanced by the current study efforts.

## Materials and methods

### Description of the study location

The study region is the urban location of Lahore, the capital of Punjab province and a major hub of trade in Pakistan, located at 31.5204°N latitude and 74.3587°E longitude. Lahore is a medieval metropolis spanning 1772 square kilometers and is located on the western bank of the River Ravi at an elevation of 217 m above sea level. Lahore is the second-largest city in Pakistan and one of the most populated cities in the world, with an estimated 12.64 million people and a growth rate of 3.83%. Lahore is the commercial hub of Punjab, where most people work in trade, industry, and commerce, hence more people are moving there from all over the country. The population of the city is gradually increasing, and the city's quick expansion and development are increasing the amount of vehicles that travel through it.

**Figure 7:**
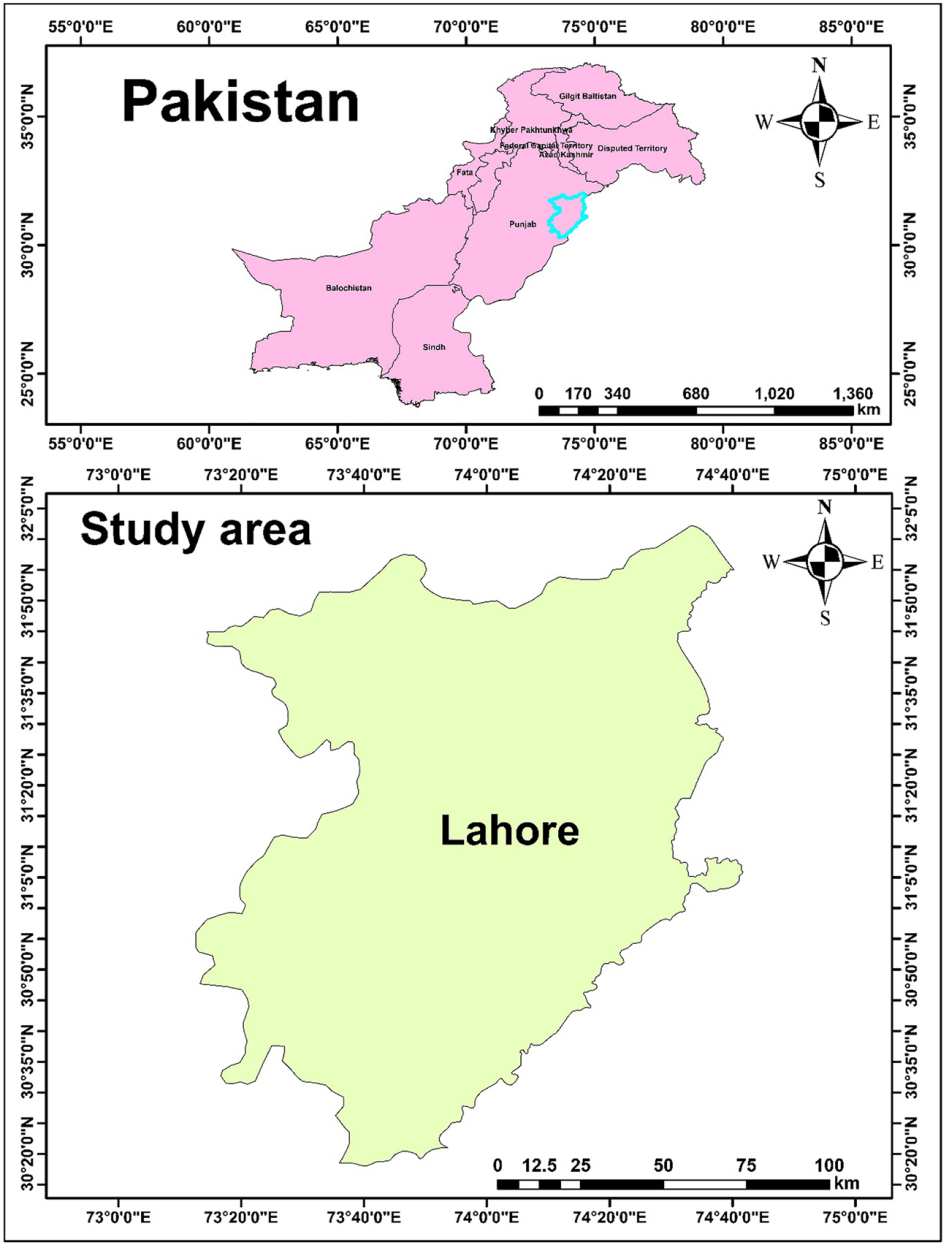
Map of the current study location i.e. Lahore, Pakistan. The map was generated using ArcMap version 10.7.1.

Paper, textiles, steel, cigarettes, chemicals, steel, textiles, construction materials, medicines, and industrial food processing are the main industries in the center of Lahore. Lahore city is the hub of offices, schools, colleges, and different kinds of residential neighborhoods, which makes it vulnerable to vehicle emissions from heavy traffic. In addition, rush-hour traffic is still bad in the morning and late afternoon as most people head home from work, school, and college (Fig. 7).

The city center experiences primarily hot weather from May to August^24,25,67^, with an average temperature of 40 °C. The monsoon season (July and September) is when a series of wet spells starts^68,69^. The winter season begins in December and lasts through February, with an average temperature of 6 °C^24,25,70^. The wintertime is typically chilly and cloudy^22^.

### Meteorological condition of the study location

**Figure 8:**
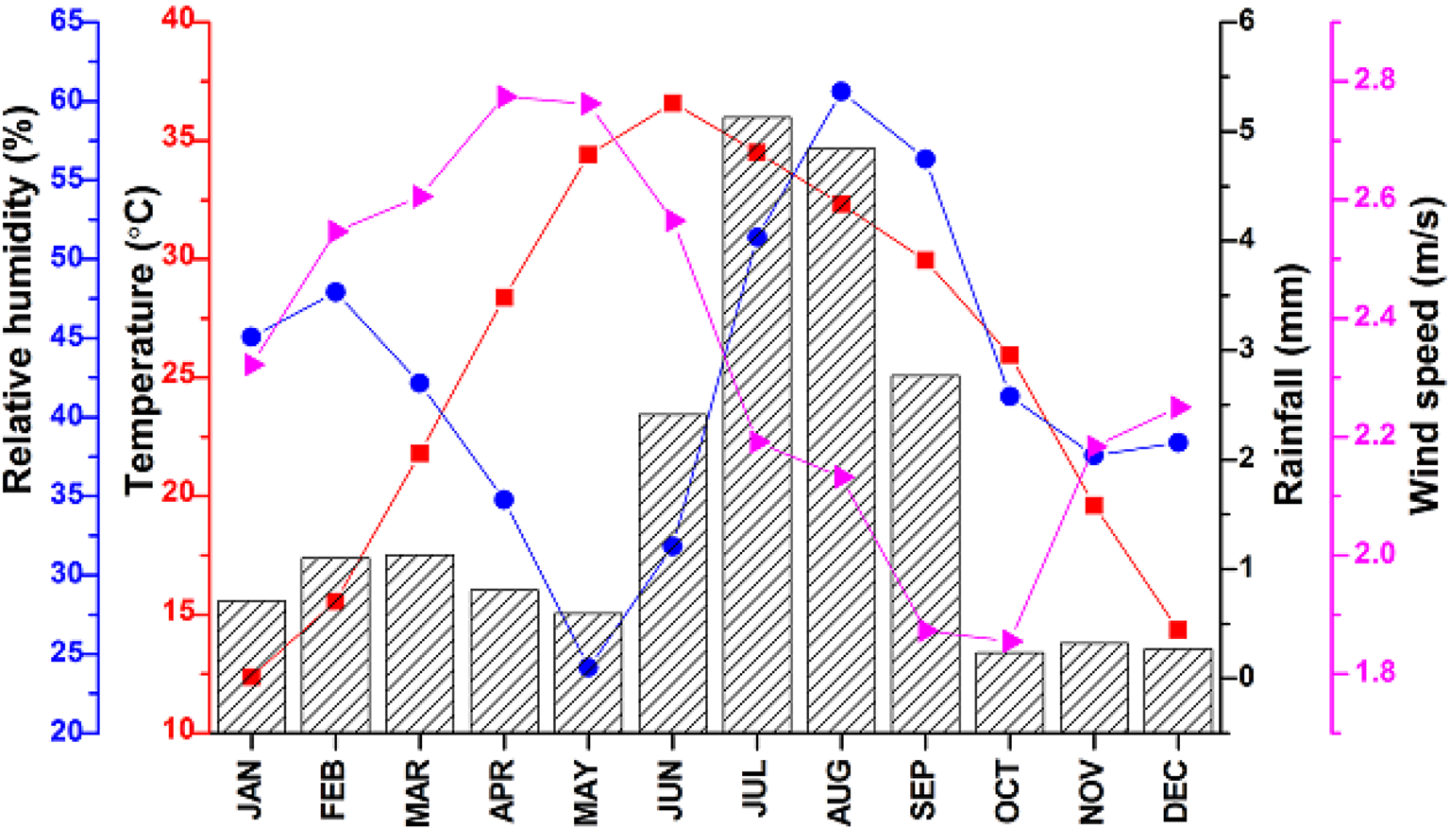
The meteorological distribution of relative humidity (%), Temperature (°C), rainfall (mm), and wind speed (m/s) during the study period from 2007 to 2020 over urban Lahore.

We collected local meteorological data from the Pakistan Meteorological Department, including relative humidity, temperature, rainfall, and wind speed. Figure 8 shows the mean monthly variation in the ambient temperature (°C), relative humidity (%), rainfall (mm), and wind speed (m/s).

Relative humidity values are observed to follow a bi-model distribution, with two peaks around 61% in July and 48% in February. The temperature can reach up to 37 °C in June. After that, it progressively drops until January, when it reaches its lowest point of 12 °C. According to the distribution, there was a significant concentration of precipitation from June to September, with a peak of 5 mm in July, while very little rainfall from October to December. The shift in the monthly variation in wind speed is observed to be quite slight, with April and May recording the highest monthly wind speed (2.8 m/s). The four seasons that are the subject of this study, are spring (March–May), summer (June–August), autumn (September–October), and winter (December–February).

### Instrumentation

#### AERONET

The Aerosol Robotic Network (AERONET) is a NASA-established network of over 400 permanent and temporary locations worldwide that is used for ground-based remote sensing of aerosols. One of the AERONET stations, Lahore was put into service in December 2006 as a result of a partnership between NASA and Pakistan Space and Upper Atmosphere Research Commission (SUPARCO), located in the Institute of Space Technology, Lahore's Space and Atmospheric Sciences Division. A CIMEL sun/sky radiometer, which measures diffuse sky radiance from 440 to 1020 nm and direct sun radiance from 340 to 1020 nm spectral ranges, is the component of an AERONET device^71^. AERONET provides aerosol data at three levels i.e. level 1.0 (unscreened), level 1.5 (cloud screened)^72^, and level 2.0 (cloud screened and quality assured)^71^. For the current study, AERONET level 2.0 (cloud screened and quality assured) has been used for the investigation of AOD, AE, perceptible water, SSA, ASY, and volume size distribution during the study 2007–2020. Level 1.0 (unscreened), level 1.5 (cloud screened)^72^, and level 2.0 (cloud screened and quality assured) are the three levels of aerosol data that AERONET offers. AOD, AE, noticeable water, SSA, ASY, and volume size distribution have all been investigated for the current study using AERONET level 2.0 (cloud screened and quality certified) during the study 2007–2020.

### Consent to participate

The authors have given their approval to take part in this study.

## Data Availability

The corresponding author is agreeable to grant the data utilized in this research work on behalf of realistic appeal.
